# Effects of Vitamin B6 Therapy for Sepsis Patients with Linezolid-Associated Cytopenias: A Retrospective Study^☆^

**DOI:** 10.1016/j.curtheres.2012.12.002

**Published:** 2013-06

**Authors:** Jie Deng, Long-xiang Su, Zhi-xin Liang, Li-ling Liang, Peng Yan, Yan-hong Jia, Xin-gang Zhang, Dan Feng, Li-xin Xie

**Affiliations:** 1 Department of Respiratory Medicine, the Chinese PLA General Hospital, Beijing, China; 2 School of Medicine, Nankai University, Tianjin, China; 3 Medical Statistics Division, the Chinese PLA General Hospital, Beijing, China

**Keywords:** linezolid, linezolid-associated cytopenias, sepsis, vitamin B6

## Abstract

**Background:**

The common adverse effects of linezolid for treating septic patients with gram-positive cocci is anemia and thrombocytopenia, which limit its clinical application.

**Objectives:**

We determined the effects of vitamin B6 adjunctive therapy on linezolid-associated cytopenias, and retrospectively studied 75 septic patients who received at least 7 days of linezolid treatment.

**Methods:**

Patients were divided into a linezolid treatment group (LTG; n = 41) that received linezolid only and a combination treatment group (CTG; n = 34) that received both linezolid and vitamin B6. Each group was further subdivided into those with sepsis and those with severe sepsis. Each patient had red blood cell (RBC), hemoglobin (Hb), hematocrit (Hct), and platelet (PLT) measurements at baseline (day 0) and every other day for 2 weeks during treatment; these parameters were compared between the groups and assessed for time-dependent trends.

**Results:**

For patients in the LTG, RBC, Hb, and Hct values showed statistically significant reductions over time, and these values were lower compared with the values in the CTG. The CTG also showed downward trends, except on the first day of treatment. The PLT count also decreased in both groups. Patients with severe sepsis had lower PLT counts in both treatment groups compared with the septic patients.

**Conclusions:**

Septic patients who received a combination treatment of linezolid and vitamin B6 might show positive effects for linezolid-associated reductions in some hematologic parameters (RBC, Hb, and Hct). This combined treatment might also slow PLT reduction, which was more evident in patients with severe sepsis. ClinicalTrials.gov identifier: NCT01295801.

## Introduction

Sepsis occurs frequently, has a high mortality rate, incurs high treatment costs, and is 1 of the leading causes of death in an intensive care unit (ICU). Thus, it is of great importance to expand the range of treatment options for patients with severe sepsis to achieve higher survival rates. The primary pathogens involved in sepsis infections are gram-positive bacteria. However, for ICU patients, the number of infections due to drug-resistant, gram-positive bacteria is increasing, especially those due to *Staphylococcus aureus*, coagulase-negative staphylococci and enterococci, and methicillin-resistant *Staphylococcus aureus* (MRSA)^1^; these infections pose a pressing problem in the treatment of patients with sepsis.

Vancomycin and linezolid are among the first treatment choices for MRSA infections.^2^ However, a number of vancomycin-resistant strains of MRSA have been reported.^1^ The high efficacy of linezolids, a new line of antibacterial drugs that was introduced within the past decade, has made valuable contributions to the treatment of vancomycin-resistant strains of MRSA.^3^ However, as linezolids have become more widely used, their adverse effects on the circulatory system,^4^ including anemia and thrombocytopenia,^5^ have become more prominent, thus limiting their clinical applications. However, Spellberg et al^6^ reported that combination therapy with vitamin B6 could reverse linezolid-induced thrombocytopenia and prevent these adverse events.

Thus, the purpose of this retrospective study was to further explore how the application of vitamin B6 as an adjuvant treatment affects linezolid-associated cytopenias in septic patients.

## Materials and Methods

### Patients

Patients for this study were selected from the respiratory care unit and the surgical ICU at the PLA General Hospital in Beijing, China, from January 2010 to March 2011. All of the patients signed an informed consent form. Patients with sepsis were divided into different groups based on either a clear or highly suspected infection focus and at least 2 of the following criteria: (1) body temperature either >38^o^C or <36^o^C; (2) heart rate >90 beats/min; (3) either respiratory rate >20 times/min or partial pressure of carbon dioxide in the arteries <32 mm Hg; or (4) peripheral blood white blood cell count >12.0 × 10^9^/L or <4.0 × 10^9^/L or immature neutrophils >10%.^7^ Severe sepsis was associated with organ dysfunction, hypoperfusion, and hypotension. Patients with severe sepsis exhibited at least 1 of the following: (1) hypoxemia (oxygenation index partial pressure of oxygen in the arteries/fraction of inspired oxygen <300); (2) acute renal injury as evidenced by either oliguria (urine output <0.5 mL/kg/h for ≥2 hours) or a serum creatinine increase of >0.5 mg/dL (44.2 μmol/L); (3) coagulation abnormalities, in which activated partial thromboplastin time was >60 seconds, international normalized ratio was >1.5 seconds, or platelet (PLT) count was <100 × 10^9^/L; (4) hyperbilirubinemia, in which total bilirubin was either >4 mg/dL or 70 mmol/L; (5) metabolic acidosis, in which either pH was <7.3 or lactic acid levels were >2 times higher than normal (lactic acidosis); and (6) infection-associated hypotension, in which systolic blood pressure was <80 mm Hg, mean arterial pressure (MAP) was <70 mm Hg, or systolic blood pressure had a decrease of >40 mm Hg.^8^

The study exclusion criteria were the following: (1) <18 years of age; (2) AIDS; (3) neutral neutropenia (polymorphonuclear neutrophil count of <500 μL^–1^); (4) linezolid treatment course of <7 days, and (5) use of chloramphenicol and thiamphenicol for treatment.

### Patient Groups and Treatments

All patients received either intravenous or oral linezolid for at least 7 days for a gram-positive bacterial infection. We included 75 patients with sepsis in this study. One group received linezolid only (linezolid treatment group [LTG]; n = 41) and another group received a combined treatment of linezolid and vitamin B6 as an adjuvant therapy (combined treatment group [CTG]; n = 34). We further subdivided the LTG and the CTG into 2 subgroups: sepsis and severe sepsis. For the CTG, there were 18 patients with sepsis (CTG1) and 16 patients with severe sepsis (CTG2). For the LTG, there were 26 patients with sepsis (LTG1) and 15 patients with severe sepsis (LTG2).

For linezolid treatment, we used either linezolid intravenous injections (300 mL [600 mg]/bag, lot number 09K20Z40; Fresenius Kabi Norge AS, Oslo, Norway) or linezolid tablets (600 mg/tablet, batch number C100082; Pfizer Pharmaceuticals LLC, New York, New York). The linezolid injection was 300 mL (600 mg) administered intravenously, and the linezolid tablets were 600 mg, taken orally once every 12 hours. For the vitamin B6 adjuvant treatment, we used an injection of amino acids (1 mL [50 mg]/support, production lot number approved by the state H12020522 products; Tianjin Jingyao Amino Acid Co, Ltd, Tianjin, China). Vitamin B6 treatment was administered at 2 mL (50 mg) by intravenous injection, to a total of 100 mg/d.^6,9^

### Study Measurements

The study was approved by the local ethics committee. For each patient, we recorded age, sex, days of drug compliance, Acute Physiology and Chronic Health Evaluation (APACHE II) scores, instances of a transfusion of blood products during treatment, underlying diseases and mortality, and other general information. We used routine clinical laboratory methods to determine red blood cell (RBCs) counts, hemoglobin (Hb), hematocrit (Hct), and PLT counts. These 4 indicators were determined before medication administration (baseline, day 0) and on days 1, 3, 5, 7, 9, 11, 13, and 15 during the treatment course.

### Statistical Methods

Data analysis was performed using SPSS (version 16.0 statistical software; IBM, Armonk, New York). Results for continuous variables are presented as means (SD) and were compared using Student’s *t*-test. Results for categorical variables are given as the number of cases (percentage) and compared using a χ^2^ test. We used repeated-measures ANOVA and independent sample *t*-tests to compare the groups for differences at different time points and by medication given before and during treatment for laboratory test result changes in RBCs, Hb, Hct, and PLT. A *P* value <0.05 was considered statistically significant.

## Results

### Patient Demographic Characteristics

Our CTG included 34 patients, and the LTG included 41 patients. As shown in Table I, patients in the CTG took their medication for 15.9 (8.2) days, whereas those in the LTG took their medication for 14.2 (6.2) days; these durations were not significantly different (*P* > 0.05). There were also no significant differences between these groups in age, sex, APACHE II scores, the number of instances of a transfusion of blood products during treatment, blood filtration conditions, underlying disease conditions, or mortality (*P* > 0.05).

### Trends in Hematology Results during the Treatment of Septic Patients

#### Red Blood Cell Counts

**Figure 1:**
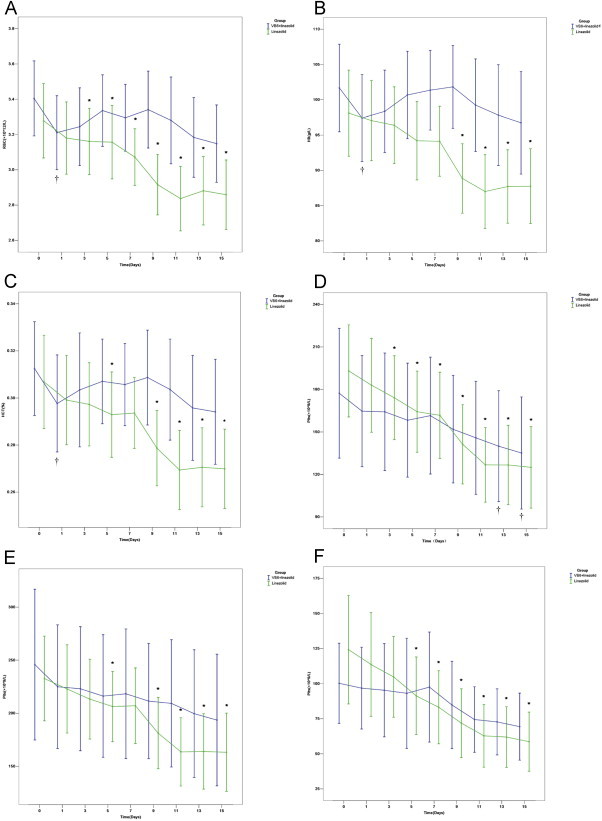
Trends in laboratory parameters with treatment time. (A) Red blood cell (RBC), (B) hemoglobin (Hb), (C) hematocrit (Hct), (D) platelet (PLT), (E) PLTs for septic patients, and (F) PLTs for severe septic patients. ^⁎^*P* < 0.05 compared with the linezolid treatment group before treatment. ^†^*P* < 0.05 compared with the combined treatment group before treatment. VB6, vitamin B6.

Figure 1A shows the trends in RBCs for septic patients in the LTG and the CTG at different times during their treatments (Table II). Compared with baseline (day 0) values, the RBC levels for septic patients in the LTG showed a significant reduction with time starting on day 3. By comparison, patients in the CTG did not exhibit any obvious downward trend in RBCs over time, except for the first day of treatment, on which their RBC levels were slightly lower than those before treatment (*P* < 0.05). Starting on day 9, the RBC results of the CTG and the LTG were significantly different (*P* < 0.05). This illustrated that patients treated with linezolid alone had a more significant downward trend in RBC levels compared with patients receiving linezolid treatment in combination with vitamin B6.

#### Hemoglobin

Figure 1B shows that treatment with linezolid alone (LTG) resulted in a significant decrease in Hb levels over time compared with the Hb levels before treatment (Table II). This slow decline became apparent on day 5. For patients in the CTG, a reduction in Hb levels was not obvious, except that the Hb levels on the first day of treatment compared with those before treatment were lower (*P* = 0.018). The Hb levels of the CTG and the LTG differed significantly from the ninth day onward, with lower Hb levels in the LTG.

#### Hematocrit

The trends for the Hct are shown in Figure 1C (Table II). Figure 1C shows that the Hct of the LTG appeared to decrease on the fifth day compared with patients in the CTG, and on the ninth day, Hct was significantly lower. However, in the CTG, a decline in Hct was not obvious, with the exception that Hct on the first day of treatment was significantly decreased compared with baseline (day 0). With regard to the Hct, patients in the LTG had a significant downward trend compared with those in the CTG.

#### Platelet Counts

As shown in Figure 1D (Table II), septic patients in both the LTG and CTG showed downward trends in their PLT counts during treatment, although this time-dependent downward trend was only statistically significant in the LTG. However, there were no significant differences between these 2 groups at the different observation times (*P* < 0.05).

#### Platelet Counts in Patients with Nonsevere Sepsis and Severe Sepsis

To further evaluate the declines in the PLT during treatment, each patient group was further divided into subgroups of nonsevere sepsis and severe sepsis. The time-dependent trends for nonsevere sepsis patients are shown in Figure 1E (Table II) and for severe sepsis patients in Figure 1F (Table II). As shown in Figure 1E for the 2 sepsis groups, there were no significant differences in the PLT count between these groups (*P* > 0.05). In the CTG, the PLT values did not statistically differ from baseline. For patients with nonsevere sepsis in the LTG, during the course of treatment, between 3 and 7 days, there was no significant difference compared with samples taken before treatment. However, starting on day 9, there was a significant downward trend in the PLT count.

As shown in Figure 1F for the 2 severe sepsis subgroups (LTG2 and CTG2), there were no statistically significant differences at any of the observation points. In the CTG, although there appeared to be a downward trend in PLTs, this was not statistically significant. For severely septic patients in the LTG, statistically significant differences from baseline were observed from the fifth day onward during the course of treatment. It should be noted that, overall, the PLT values were lower for severely septic patients (Figure 1F) compared with patients with nonsevere sepsis (Figure 1E) regardless of the treatment regimen.

## Discussion

Sepsis is a condition caused by systemic inflammation and is 1 of the leading causes of death in the ICU. Although in-depth studies have been conducted on both the pathogenic mechanisms of sepsis and its pathophysiology in recent years and more treatments have been discovered, the high morbidity and mortality associated with infection remain difficult to counteract.^10^ In an epidemiological survey of surgical ICUs in 10 teaching hospitals located in 6 Chinese provinces, 53.8% of patients were treated for gram-positive infections, 45.9% of patients were treated for gram-negative bacterial infections, and 22% of patients were treated for invasive fungal infections.^11^ Gram-positive strains were the major pathogenic strains associated with sepsis.

Linezolid was the first oxazolidinone antibiotic to be approved for treating gram-positive bacterial infections.^3^ It not only exhibits excellent therapeutic effects against multiple resistant gram-positive bacteria such as vancomycin-resistant *Enterococcus faecalis*, MRSA, and multidrug-resistant strains,^12^ but it also has antibacterial activities against anaerobic bacteria and atypical mycobacteria.^13,14^ However, as the use of linezolid has become more widespread in clinical practice, several adverse outcomes have been observed in patients who received long-term linezolid treatments.

After using the drug for >2 weeks, adverse outcomes primarily affecting the hematopoietic system were reported, including anemia and thrombocytopenia.^4^ In a study of disseminated mycobacterial infections in which patients received both linezolid and vitamin B6, Spellberg et al^5^ observed that vitamin B6 might reduce or even prevent linezolid-related thrombocytopenia. Subsequently, in a retrospective study of 24 patients with bone marrow infections who received both linezolid and vitamin B6, the opposite conclusion was found, in that adjuvant treatment with vitamin B6 for sepsis in combination with linezolid did not reduce linezolid-related hematologic adverse outcomes.^9^ In a study by Youssef et al,^15^ who used linezolid and vitamin B6 as an adjuvant therapy for 31 cancer patients complicated by infections, vitamin B6 reduced secondary anemia, but it did not prevent the reduction in PLT and leukocyte counts.

Our retrospective clinical study found that patients who were treated for sepsis with linezolid had reductions in RBC counts, Hb, Hct, and PLT counts. On the first day of observation for the linezolid and vitamin B6 treatment group, the RBC, Hb, and Hct levels were significantly lower than baseline, but throughout the remainder of the treatment course used in this study the downward trend observed just after treatment began did not significantly change. Septic patients who received vitamin B6 combined with linezolid also showed similar effects for PLT counts, although the downward trend in PLTs was only statistically significant after the first 13 days of treatment. Thus, it could be inferred that vitamin B6 used in adjuvant therapy could prevent linezolid-related reductions in blood cells to some extent, and although it was highly effective for red cells, its effects on thrombocytopenia might not be as obvious.

Hb is comprised of heme and globin, and vitamin B6 is essential for hemoglobin synthesis. It participates in the synthesis of a heme precursor, -δ-amino-γ-keto acid, and is a δ-amino-γ-keto acid synthesis enzyme cofactor.^16^ Sideroblastic anemia is defined as a vitamin B6 genetic factor heme synthesis pathway disorder. This Hb gene mutation may occur in cell anemia, small cell anemia, and megaloblastic anemia; thus, vitamin B6 treatment is useful for such patients.^17^ Additionally, linezolid inhibits mitochondrial protein synthesis and blocks mitochondrial respiration, which may cause sideroblastic anemia, because RBCs produce an important factor to counteract this effect.^18^

In our CTG, the RBCs, Hb, and Hct were only significantly lower on the first day after starting treatment. Concurrently, the LTG exhibited significant reductions in the RBC counts over time, indicating that linezolid might cause a temporary RBC gene mutation and temporarily inhibit mitochondrial respiration, which would result in this type of anemia in these patients. Vitamin B6 could promote the synthesis of hemoglobin, which could restore the RBCs in the later periods of treatment and maintain the levels observed before treatment. It could be inferred that linezolids might play a critical role by affecting RBCs and reducing the RBC pigment metabolic pathway.

One report suggested that linezolid treatment-induced thrombocytopenia might be immune-mediated,^19^ which was similar to the mechanism associated with quinine and/or quinidine-like immune-mediated thrombocytopenia. This immune-related thrombocytopenia is due to either a drug or its metabolites binding to either the PLT membrane glycoprotein 1b/IX or glycoprotein IIb/IIIa, which produces an immunogenic compound of immunoglobulin-G and a Fab fragment. Immunoglobulin-G and an Fc fragment can combine with macrophages and be cleared by the reticuloendothelial system, resulting in thrombocytopenia.^20,21^

In this study, patients treated with both linezolid and vitamin B6 did show changes in PLTs during the first 9 days of medication use, but these changes in PLTs were not statistically significant until 13 days after initiation of treatment compared with the samples taken before treatment began. In the sepsis subgroups CTG1 and LTG1, there was a downward trend despite the fact that the CTG and 2 patients in the LTG did not show significant differences at each observation point. In particular, the decline observed in the CTG was obviously slower. In the severe sepsis subgroups CTG2 and LTG2, the downward trend in the group treated with linezolid alone was clearly more evident, although the PLT counts were not significantly different at each observation point. We speculated that vitamin B6 given to septic patients with linezolid-related thrombocytopenia might slow the loss of PLTs, but this was not conclusive.

It was reported that linezolid blood levels might affect hematopoietic function and that clearance of linezolid was closely related to Hb levels.^22^ We speculated that this latter mechanism involved alterations in PLT dynamics by vitamin B6. This might due to the maintenance of Hb levels, which could increase the clearance of linezolid and reduce its accumulation in the body, thus reducing the chance of immune complex formation when linezolid binds to either the membrane glycoprotein 1b/IX or glycoprotein IIb/IIIa. Thus, there would not be a significant reduction in PLT counts. However, with an extended treatment time, there was a clear reduction in the PLT count, which suggested that there was no significant effect on the ability of vitamin B6 to reduce PLT counts.

This retrospective study had some limitations. First, this was a single-center retrospective study; thus, the experimental controls were not ideal. Second, because the overall sample size was small, the power of the statistical tests might have been reduced, so the probability of significant reductions might have been low. Third, the patients only received medication for approximately 14 days, which might have caused some limitations with regard to medication use duration. Finally, this study used both oral and intravenous administration; the route of administration for different drugs could affect the occurrence of adverse reactions because these depend on bioavailability and pharmacokinetic parameters.^23^ We could speculate that the bioavailability and pharmacokinetic parameters of intravenously infused linezolid might be different from those of orally administered linezolid. In this retrospective study, we did not distinguish between intravenous and oral administration. Thus, we could not rule out that a different route of linezolid administration might reduce the observed effects on the measured blood cell parameters.

In summary, patients who received linezolid for extended periods could develop linezolid-related cytopenias. Thus, before and during treatment, we should closely monitor changes in hematologic parameters. Care should be taken during symptomatic treatment to stop administration of the medicine when reductions in blood cell counts are noted. In this preliminary retrospective study, we found that administering linezolid, along with adjuvant treatment with vitamin B6, to patients with sepsis either prevented or delayed the reductions in the RBC counts to a certain extent, whereas no improvement was observed in the development of thrombocytopenia. Vitamin B6 is a low-cost supplement with few side effects. Thus, its use in conjunction with linezolid could be regarded as an effective method to reduce blood cell counts. These observations will require either additional studies with larger sample sizes or a prospective study to confirm the effects noted here. Furthermore, studies must also be conducted regarding the mechanisms involved with vitamin B6 to prevent and treat linezolid-related pancytopenia.

## Conflicts of Interest

The authors have indicated that they have no conflicts of interest regarding the content of this article.

## Figures and Tables

**Table I:** Clinical characteristics of septic patients receiving various treatments.

Variable		Linezolid + Vitamin B6 (n = 34)	Linezolid (n = 41)	*P*
Age, y, mean (SD)	56.8 (21.9)		61.8 (18.5)	0.082
Gender, n (%)				0.395
Male	20 (68.8)		28 (68.3)	
Female	14 (41.2)		13 (31.7)	
Duration of linezolid, d, mean (SD)	15.9 (8.2)		14.2 (6.2)	0.379
APACHE II, mean (SD)	15.7 (5.8)		19.2 (7.4)	0.194
Blood product transfusion, n (%)	22 (64.7)		24 (58.5)	0.585
Hemofiltration, n (%)	6 (17.6)		5 (12.2)	0.506
Underlying disease				
Hypertension , n (%)	7 (20.6)		11 (26.8)	0.529
COPD, n (%)	4 (11.8)		6 (14.6)	0.716
Chronic renal disease, n (%)	8 (23.5)		11 (26.8)	0.744
Diabetes, n (%)	3 (8.8)		8 (19.5)	0.193
Immune suppression, n (%)	4 (11.8)		4 (9.8)	0.779
Nervous system disease, n (%)	5 (14.7)		5 (12.2)	0.698
Coronary heart disease, n (%)	11 (32.4)		14 (34.1)	0.870
No underlying disease	10 (29.4)		7 (17.1)	0.209
Death, n (%)	14 (41.2)		13 (31.7)	0.312

APACHE, Acute Physiology and Chronic Health Evaluation; COPD, chronic obstructive pulmonary disease.

**Table II:** Repeated measures of the ANOVA results for red blood cell counts (RBCs), hemoglobin (Hb), hematocrit (Hct), and platelet counts (PLTs).

Time Points of Observation, d	RBCs		*P*	Hb		*P*	Hct		*P*	PLTs		*P*	PLTs in Nonsepsis Patients		*P*	PLTs in Severe Sepsis Patients		*P*

LTG	CTG	LTG	CTG	LTG	CTG	LTG	CTG	LTG	CTG	LTG	CTG

0⁎	3.29 (0.71)	3.34 (0.59)	0.751	98.10 (19.31)	101.68 (17.77)	0.410	0.31 (0.06)	0.31 (0.06)	0.505	193.05 (102.88)	177.32 (131.27)	0.563	232.77 (98.60)	245.83 (142.67)	0.721	124.20 (69.80)	100.25 (53.58)	0.291
1st	3.18 (0.67)	3.18 (0.58)	0.995	97.05 (18.06)	97.41 (17.68)	0.930	0.30 (0.06)	0.30 (0.06)	0.831	183.78 (107.01)	155.82 (112.63)	0.470	222.92 (102.79)	225.00 (117.22)	0.951	113.60 (66.65)	96.81 (54.60)	0.448
3rd	3.16 (0.63)	3.18 (0.54)	0.841	96.39 (17.24)	98.35 (16.14)	0.620	0.30 (0.06)	0.30 (0.07)	0.356	174.17 (93.57)	164.21 (118.82)	0.686	214.19 (89.13)	225.44 (126.66)	0.728	104.80 (51.97)	95.31 (62.33)	0.650
5th	3.09 (0.69)	3.29 (0.50)	0.168	94.20 (17.61)	100.68 (17.76)	0.118	0.29 (0.06)	0.31 (0.05)	0.117	164.29 (90.65)	158.29 (115.26)	0.802	206.39 (81.96)	216.22 (116.00)	0.743	91.33 (49.90)	93.13 (73.63)	0.938
7th	3.10 (0.62)	3.27 (0.50)	0.163	94.12 (15.69)	101.35 (16.18)	0.054	0.29 (0.05)	0.31 (0.05)	0.130	161.76 (96.32)	161.53 (118.26)	0.993	207.12 (88.08)	218.39 (122.73)	0.724	83.13 (47.00)	97.56 (73.51)	0.523
9th	2.95 (0.58)	3.31 (0.56)	0.008	88.85 (15.58)	101.82 (16.82)	0.001	0.28 (0.05)	0.31 (0.06)	0.006	141.27 (88.63)	151.88 (108.61)	0.643	181.31 (83.16)	211.50 (109.14)	0.304	71.87 (44.24)	84.81 (58.21)	0.493
11th	2.88 (0.63)	3.26 (0.66)	0.015	87.00 (15.58)	99.24 (18.76)	0.004	0.27 (0.05)	0.30 (0.06)	0.004	126.68 (83.35)	145.85 (114.17)	0.406	163.65 (79.60)	209.33 (120.56)	0.137	62.80 (40.30)	74.44 (43.90)	0.449
13th	2.88 (0.63)	3.18 (0.65)	0.042	87.71 (16.48)	97.82 (20.47)	0.020	0.27 (0.05)	0.30 (0.06)	0.037	126.68 (88.60)	139.94 (112.03)	0.569	164.04 (87.95)	199.67 (120.90)	0.264	61.93 (39.10)	72.75 (44.16)	0.477
15^th^	2.86 (0.62)	3.15 (0.63)	0.050	87.76 (16.81)	96.74 (20.81)	0.042	0.27 (0.05)	0.29 (0.06)	0.046	125.00 (91.23)	135.15 (113.48)	0.669	163.27 (91.33)	193.61 (124.65)	0.356	58.67 (38.06)	69.38 (44.79)	0.480

*P* values represent the statistical differences between two groups at various time points of observation. CTG, combined treatment group; LTG, linezolid treatment group.

⁎ 0 indicates the initial day before treatment.
